# Effectiveness and safety of massage in the treatment of restless legs syndrome: A protocol for systematic review and meta analysis

**DOI:** 10.1097/MD.0000000000023239

**Published:** 2020-11-25

**Authors:** Shasha Hu, Xingwei He, Yajing Zhang, Songfeng Hu, Fan He, Fenfen Zhao, Qin Zhang, Tingping Liu, Changkang Wang

**Affiliations:** a College of Acupuncture-Moxibustion and Tuina, Jiangxi University of Traditional Chinese Medicine; b The Affiliated Hospital of Jiangxi University of Traditional Chinese Medicine, Nanchang, China.

**Keywords:** massage, protocol, restless legs syndrome, systematic review

## Abstract

**Background:**

Restless legs syndrome (RLS), known as a kind of neurological disease, is prevalent but easy to be ignored. Studies have demonstrated that massage therapy can effectively reduce the symptoms of patients with RLS and improve their quality of life. However, the efficacy of massage therapy for RLS is still controversial. Therefore, this protocol aims to evaluate the reliability of massage therapy in treating RLS in a thorough way.

**Methods:**

We will search relevant randomized controlled trials from Chinese Biomedical Literature Database, Chongqing VIP, CNKI, Wanfang, Web of Science, Cochrane Library, PubMed, and EMBASE, when publication status and language are not considered and the time limit ends with September 6, 2020. Two experienced researchers will use RevMan V.5.3 software to perform the selection of literature, data collection, data analysis and synthesis separately. Besides, the quality of trials involved in this study will be measured with the Cochrane bias risk assessment tool.

**Results:**

This protocol will be applied to carry out a systematic evaluation of the massage therapy purposed to treat RLS for its effectiveness and safety.

**Conclusion:**

The review will provide a credible evidence suggesting whether massage therapy is a reliable intervention for RLS.

**INPLASY registration number:**

INPLASY202090038

## 1 Introduction

Known as Ekbom disease (EKD), Restless legs syndrome (RLS) represents a form of sensorimotor dysfunction, and threatens the health of people across the world. According to the International Restless Leg Syndrome Research Group, the incidence of RLS ranges from 2 to 15% in the general population and roughly 20% to 80% in hemodialysis patients.^[1,2]^ RLS is characterized by a resistless urge to move such body parts as legs, together with abnormal sensation and potentially a sense of pain.^[3,4]^ Meanwhile, similar symptoms may be manifested in other body parts if the illness persists.^[5]^ Studies have shown that RLS patients may experience anxiety, depression, severe sleep deprivation, and the reduction to quality of life (QOL), thus placing a heavy burden on patients and the entire society.^[6–9]^ In addition, there is growing evidence suggesting that RLS may increase the risk of death among the patients with cerebrovascular and cardiovascular diseases.^[4,10,11]^ Moreover, it is more likely for pregnant women to suffer RLS, which may have an adverse effect on the neonatal outcomes.^[12–14]^ Therefore, it is of important clinical and public health significance to find more effective approaches to treating RLS.

Currently, drug therapy is the main treatment of RLS, playing an important role in reducing the symptoms of RLS.^[15]^ First-line treatments for RLS include DA drugs and α2δ agonists. However, these drugs have a potential to cause a range of evident complications, including drowsiness, headaches, gastrointestinal symptoms and so on.^[6,8,16,17]^ It is also reported that less than 15% of the patients are fit for medication treatment.^[18]^ So it is absolutely necessary to find a non-drug therapy that is more affordable, safer and effective in reducing the occurrence of side effects. Nowadays, massage therapy has been widely adopted in various clinical applications as a major Complementary and Alternative Medicine intervention.^[19]^ Furthermore, a report has been published to suggest that as many as 76.9% of RLS patients take enjoyment from having their legs massaged.^[20]^

Massage is a traditional therapy in which the hands work on the tissues of the body, including tuina, acupressure, and the likes.^[21]^ It is beneficial to the well-being of patients both physically and mentally. On 1 hand, massage can alleviate the tension in their skin and muscles, thus promoting blood circulation and enhancing the immune system.^[22]^ On the other hand, massage can prompt the releases of dopamine inside human body, thus making the patient feel pleased and relaxed.^[23]^ Plenty of research has also demonstrated that massage is effective in reducing the symptoms associated with RLS.^[20,23–26]^ Apart from that, it is an increasingly common practice among medical professionals to prescribe massage therapy due to its safety, simplicity, affordability and the prospect of reduced side effects.^[27]^

Although massage therapy has been widely used clinically and its benefits have been proven, the efficacy of massage therapy in the treatment of RLS arouses controversy. Under this context, the present systematic review will be conducted to examine the reliability of massage therapy for RLS by using the method of evidence-based medicine.

## 2 Methods

### 2.1 Study registration

The registration number is INPLASY202090038. Adhering to this agreement, we will follow the Preferred Reporting Items set out in the guidelines for the Systematic Review and Meta-Analysis Protocol (PRISMA-P) statement.^[27]^

### 2.2 Inclusion criteria for study

#### 2.2.1 Type of studies

It will include all randomized controlled trials (RCTs) about the massage intended for RLS patients while language and publication status are not considered. However, animal trials, case reports, Non-RCTs, experience reports, and reviews will not be enrolled.

#### 2.2.2 Types of participants

It will enroll all patients with restless legs syndrome, irrespective of their ethnicity, gender, and age.

#### 2.2.3 Types of interventions

##### 2.2.3.1 Experimental interventions

The experimental group will be restricted to receiving massage therapies, including tuina, acupressure, foot reflexology, etc. The frequency, strength, time, sites, and types of massage therapy are not restricted.

##### 2.2.3.2 Control interventions

Any therapies that have excluded massage will be applied to the control group, such as drugs, near-infrared light therapy, acupuncture, psychotherapy, placebo, etc.

#### 2.2.4 Types of outcome measures

##### 2.2.4.1 Primary outcomes

The visual analog scale, The International RLS Study Group Rating Scale, or other scales will be included in the primary outcomes.

##### 2.2.4.2 Secondary outcomes

The adverse events, quality of life measures, sleep disturbance, anxiety, and depression scales will be included in the secondary outcomes.

### 2.3 Search methods

#### 2.3.1 The primary source of data

The RCTs related to the massage treatment received by RLS patients will be retrieved from all available databases from their inception to September 6, 2020, including Chinese Biomedical Literature Database, Chongqing VIP, CNKI, Wanfang, Web of Science, Cochrane Library, PubMed, and EMBASE. Table 1 details the retrieval strategy adopted for PubMed.

**Table 1:** Search strategy for PubMed database.

Number	Search items
#1	randomized controlled trial [pt]
#2	controlled clinical trial [pt]
#3	randomized [tiab]
#4	clinical trials as topic [mesh: noexp]
#5	randomly [tiab]
#6	trial [ti]
#7	OR/#1–#6
#8	animals [mh] NOT humans [mh]
#9	#7 NOT #8
#10	Restless legs syndrome[Mesh]
#11	Restless leg syndrome[All Fields)
#12	Restless legs[All Fields)
#13	Periodic leg movement[All Fields)
#14	Willis Ekbom[All Fields)
#15	Wittmaack-Ekbom Syndrome[All Fields)
#16	OR/#10–#15
#17	Massage[Mesh]
#18	Massage Therapy[All Fields)
#19	Zone Therapy[All Fields)
#20	Acupressure[All Fields)
#21	Manipulate[All Fields)
#22	Tuina[All Fields)
#23	Anmo[All Fields)
#24	OR/#17–#23
#25	#9 AND #16 AND #24

#### 2.3.2 Search of other resources

Some unfinished or unpublished experimental data will be retrieved from the Chinese Clinical Trial Registry and The Clinicaltrials.gov.

### 2.4 Data collection and analysis

#### 2.4.1 Literature selection

**Figure 1:**
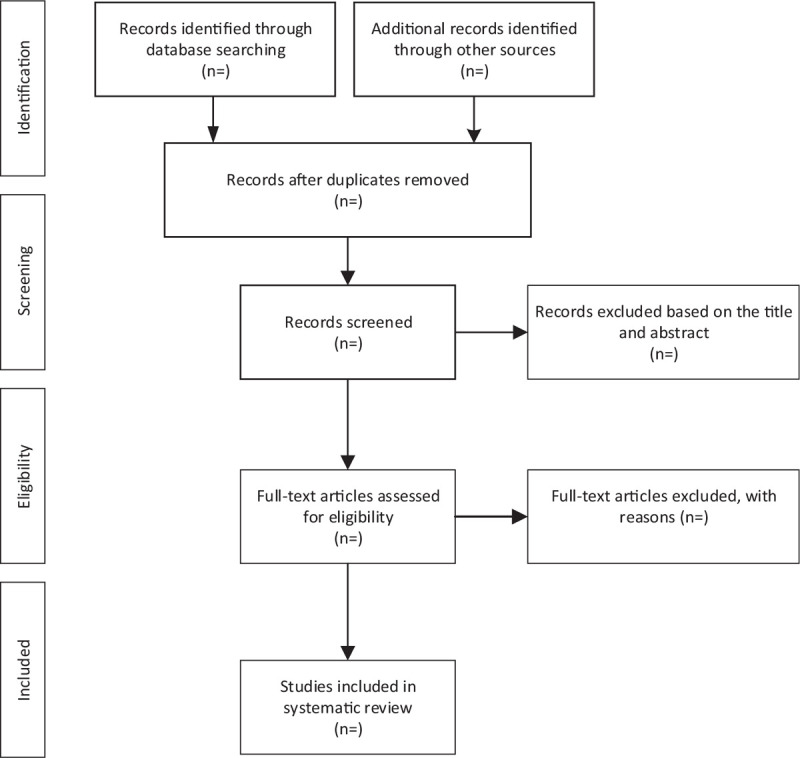
Flow diagram of study selection process.

Firstly, all literature will be iuputted into EndNote X9 software to remove all duplicate literature. Secondly, to remove the irrelevant literature, the title and abstract will be reviewed by Researcher SQ and YX. Thirdly, the whole text will be read by them to determine whether the researches will be involved in this project. Finally, 2 researchers (SQ and YX) will conduct cross-check. In case of any disagreements, a third researcher (LJ) will be present to conduct discussion to resolve them. Figure 1 shows the flowchart of literature screening.

#### 2.4.2 Data extraction and management

The 2 authors will extract qualified data separately, and a third author will intervene to resolve any potential discrepancies. The extracted data covers journal, author information, title, publication time, participant characteristics, sample size, interventions, research methods, primary, and secondary outcome measures, and any adverse events.

### 2.5 Risk of bias assessment

SQ and YX will use the Cochrane Bias risk assessment tool^[28]^ separately to examine the quality of trials involved. The extracted details include random sequence generation, result evaluation blindness, participant and personnel blindness, allocation concealment, selective result reporting, incomplete result data, etc, which are divided into several levels, including ambiguous, low and high. The author of an ambiguous research-related project will be contacted. In case of any dispute, a sensible decision will be made under the assistance of a third investigator (LJ).

### 2.6 Data synthesis

Data analysis will be conducted with RevMan 5.3 software. The risk ratio (RR) with a 95% confidence interval (CI) will be adopted when the measured outcomes are dichotomous variables. In the case of continuous variables as the measured outcomes, when the measurement tools are the same, the Weighted Mean Difference (WMD) with 95% CI will be selected; otherwise, the Standardized Mean Difference (SMD) with 95% CI will be applied.

### 2.7 Heterogeneity assessment

The level of statistical heterogeneity will be measured by the *x*^2^ test and *I*^2^ test. The research results will be considered heterogeneous when *P* > .1 and *I*^2^ ≤ 50% are not met. Otherwise, the results will not be considered as heterogeneous.

### 2.8 Subgroup analysis

When the involved tests reveal a significant level of heterogeneity, a subgroup analysis will be conducted with such factors taken into account as massage mode, the severity of restless leg syndrome, the course of disease, sample size, and so on.

### 2.9 Sensitivity analysis

The low-quality tests will be excluded by sensitivity analysis, thus ensuring the stability and reliability of the conclusions drawn from the meta-analysis.

### 2.10 Assessment of reporting biases

The test of publication bias requires funnel plot analysis when the number of RCTs exceeds 10. In addition, the causes of publication bias will be investigated by the Egger test if there are asymmetric funnel plots.

### 2.11 Quality of evidence

Two researchers will be present to measure the evidence quality of outcome indicators using Grade profiler 3.6 software, with the evaluation scale involving high Grade evidence, intermediate evidence, low evidence and very low evidence.

### 2.12 Ethics and dissemination

This research requires no ethical approval as it is not related to the personal details of patients. This protocol's results are expected to be published in a peer-reviewed journal.

## 3 Discussion

Restless legs syndrome as a kind of sensorimotor dysfunction has a high incidence, presenting threats to the health of patients. At present, drug therapy is taken as the major solution to treating RLS effectively, but they could lead to various side effects.^[6,8,16,17]^ As a commonly used complementary and alternative medicine therapy, massage can be effective in reducing the symptoms associated with RLS and improving their quality of life.^[20,23–26]^ In addition, it shows such advantages as cheap affordability, safety, the simplicity of operation and the reduction to side effects.^[27]^ Therefore, massage therapy is highly recommended by RLS patients.^[20]^ Up to now, however, the efficacy of massage therapy in the treatment of RLS remains controversial. Thus, it is hoped that this research will offer sufficient evidence and medical reference for the treatment of RLS by massage.

As far as we know, there may be some potential weaknesses in this study. Firstly, significant heterogeneity may exist due to the differences in the massaged body parts, intensity, frequency and time during the course of massaging. Secondly, the reliability of this review is related to the methodological quality and comprehensiveness of the research involved in the present protocol.

## Author contributions

**Conceptualization**: Shasha hu, Xingwei He.

**Data curation**: Shasha Hu, Yajing Zhang.

**Formal analysis**: Shasha Hu, Xingwei He.

**Funding acquisition**: Xingwei He.

**Methodology**: Shasha Hu, Yajing Zhang.

**Software**: Shasha Hu, Yajing Zhang.

**Supervision**: Xingwei He.

**Writing – original draft**: Shasha Hu, Xingwei He.

**Writing – review & editing**: Xingwei He.
